# How May the Public School Be Helpful in the Prevention of Tuberculosis

**Published:** 1904-12

**Authors:** 


### HOW MAY THE PUBLIC SCHOOL BE HELPFUL IN THE PREVENTION OF TUBERCULOSIS?

Knopf (*New York Medical Journal*, September 3, 1904,) subdivides his subject into a number of topics—the selection of the site of the school; its construction; the proper arrangement of the course of study so as not to overwhelm the child with too much work; the proper instruction of the teachers to be sure that the ideas of the board in this latter regard are being carried out; the duties of the school physician. The latter should make a regular systematic examination of the children under his charge, and should pay special attention to the sanitary condition of the school building. Knopf urges the construction of public school sanatoria for the care of tubercular and scrofulous children. To every child is given a copy of a set of rules, which the children are urged to take home to their parents. These rules are:

Do not spit, except in a spittoon or a piece of cloth or a handkerchief used for that purpose alone. On your return home have the cloth burned by your mother, or the handkerchief put in water until ready for the wash.

Never spit on a slate, floor, sidewalk or playground.

Do not put pins in your mouth.

Do not put your fingers into your mouth.

Do not pick your nose or wipe it on your hand or sleeve.

Do not wet your fingers in your mouth when turning the leaves of books.

Do not put pencils into your mouth or wet them with your lips.

Do not hold money in your mouth.

Do not put anything into your mouth except food and drink.

Do not swap apple-cores, candy, chewing-gum, half-eaten food whistles, bean-blowers, or anything that is put in the mouth.

Peel or wash your fruit before eating it.

Never cough or sneeze in a person's face. Turn your face to one side, and hold your hand or handkerchief before your mouth.

Keep your face and hands and fingernails clean; wash your hands with soap and water before each meal.

When you don't feel well, have cut yourself, or have been hurt by others, do not be afraid to report to the teacher.—*Cincinnati Lancet-Clinic.*

